# Clear aligner therapy beyond esthetics: oral health, polymer materials, and the environmental cost of digital orthodontics

**DOI:** 10.3389/fdmed.2026.1810940

**Published:** 2026-06-09

**Authors:** Javier Iván Martínez-Hernández, Carlos Esteban Villegas-Mercado, Sandra Aidé Santana-Delgado, Grissel Guadalupe Orozco-Molina, Juan Antonio Arreguín-Cano, Karina Ordóñez-Torres, Manuel Antonio Luján-Aguilar, Laura Isabel Duarte-Chávez, Adolfo González-Acosta, Cecilia Casavantes-Lazo, Claudia Ivette Bujanda-Ríos, Mercedes Bermúdez

**Affiliations:** 1 Faculty of Dentistry, Autonomous University of Chihuahua, Chihuahua, Mexico; 2 Anáhuac University Querétaro, Querétaro, Mexico

**Keywords:** clear aligner therapy, digital orthodontics, environmental sustainability, life cycle assessment (LCA), microplastics, oral microbiome, orthodontic aligners, polymer-based biomaterials

## Abstract

Clear aligner therapy (CAT) has become a popular orthodontic option, driven by advances in digital workflows, increasing aesthetic demands, and perceived benefits in comfort and oral hygiene. Although its clinical effectiveness has been well documented, a thorough review of its biological and environmental effects remains incomplete. This narrative review consolidates current evidence on CAT, exploring their history, material makeup, impact on oral health, and emerging environmental concerns. Recent clinical and microbiological research indicates that CAT may improve plaque control and periodontal health compared with fixed appliances; however, these benefits are heavily influenced by patient behavior, baseline caries risk, treatment duration, and adherence to hygiene and dietary guidelines. Evidence shows that aligner materials can support bacterial and fungal biofilm growth and, under certain conditions, may lead to enamel demineralization or erosion, emphasizing the need for personalized risk assessments and proper aligner maintenance. From a materials standpoint, modern aligners are primarily made from thermoplastic and polyurethane polymers, designed to exert controlled orthodontic forces and ensure durability. While beneficial clinically, these materials are poorly degradable and have limited recyclability. Life cycle analyses reveal that the environmental impact of CAT extends beyond disposal to include polymer production, energy-intensive manufacturing, packaging, and distribution. Additionally, emerging research suggests that aligners may release microplastics during use, adding to concerns about plastic pollution beyond solid waste. Given the rapid growth of the global clear aligner market, even small amounts of material per patient can result in a significant environmental impact. Overall, the evidence indicates that clear aligner therapy involves a complex interplay among biomechanics, patient care, materials science, and environmental sustainability. An integrated, life–cycle–based strategy is crucial to guiding clinicians, researchers, and manufacturers toward treatments that are both effective and environmentally responsible.

## 1 Introduction

Fixed orthodontic treatment remains one of the most widely used approaches for intercepting, correcting, or preventing malocclusions; however, it also creates retentive sites that make biofilm control more difficult and can favor gingival inflammation, enamel demineralization, and dysbiotic changes in the oral microbiota (1–4). In response to the growing demand for more aesthetic, comfortable, and less disruptive alternatives, CAT has expanded rapidly and is now commonly framed as part of contemporary digital orthodontics (5–8). This expansion is clinically relevant, not only because aligners are removable and visually discreet, but also because recent evidence suggests they may facilitate oral hygiene maintenance and support more favorable periodontal conditions than fixed appliances in selected patients (9–11).

From a materials perspective, CAT relies on the sequential use of transparent thermoplastic trays produced from digital treatment planning, commonly by thermoforming over printed models or, increasingly, by direct 3D-printing workflows (6, 7). Commercial aligners are mainly fabricated from thermoplastic polymers such as polyurethane- and polyester-based materials, chosen for their transparency, elasticity, dimensional stability, and force-delivery behavior (12). Nevertheless, these materials do not remain unchanged in the oral cavity. Saliva, thermal fluctuations, insertion–removal cycles, masticatory loading, beverages, cleaning procedures, and time-dependent surface wear can modify their chemical, optical, and morphological properties, including thickness, roughness, transparency, and stress behavior (13, 14). This intraoral aging process is clinically important because tribological wear and surface degradation may alter aligner performance and also influence microbial adhesion and biofilm accumulation on the device surface (15). Therefore, a clinically meaningful understanding of CAT requires not only understanding what aligners are made of, but also how those polymers behave under real or simulated in-mouth conditions.

**Figure 1:**
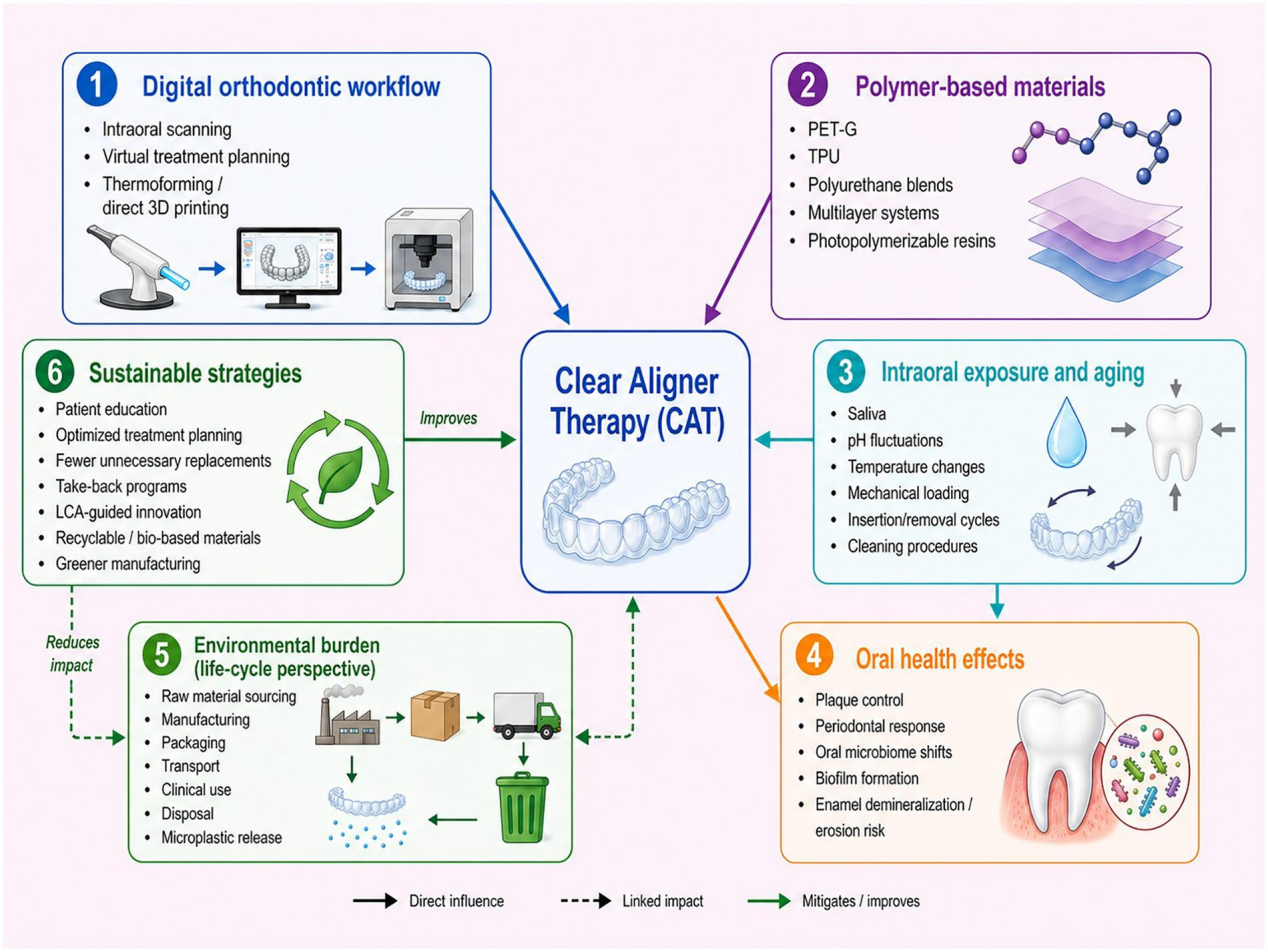
Conceptual framework linking clear aligner therapy, oral health, polymer materials, and environmental sustainability. Clear aligner therapy is based on digital workflows and thermoplastic or photopolymerizable materials. During clinical use, aligners are exposed to saliva, pH fluctuations, thermal changes, mechanical loading, cleaning procedures, and microbial colonization. These factors may influence material aging, biofilm formation, oral microbiome dynamics, enamel demineralization risk, and microplastic release. From an environmental perspective, clear aligner therapy should be evaluated through a life-cycle framework that includes raw material sourcing, manufacturing, packaging, transport, clinical use, disposal, and potential recovery or recycling strategies

This materials-based perspective also links CAT to a broader environmental discussion. Because treatment requires the serial replacement of multiple short-life polymer trays, aligners contribute to the growing stream of polymer-dependent products used in oral healthcare (16–18). Importantly, the environmental burden of CAT is not limited to discarded trays; it also involves raw material sourcing, manufacturing, packaging, transport, and the still-limited end-of-life options available for saliva-contaminated thermoplastics (17, 19). In parallel, recent evidence indicates that aligners may release microplastic particles during use under simulated or clinically relevant conditions, likely due to mechanical stress, cyclic loading, and polymer surface degradation (20, 21). This shifts the discussion from a simple “plastic waste” narrative toward a more comprehensive framework that includes life-cycle thinking, in-use microplastic generation, and practical mitigation strategies such as reducing unnecessary replacements, improving packaging and logistics, and promoting more sustainable materials and recovery pathways (16, 18). Therefore, the purpose of this review is to synthesize current evidence on what CAT are, the polymers from which they are manufactured, how these materials behave in the oral environment, and the clinical and environmental implications of CAT, including both life-cycle burden and microplastic release during use (Figure 1).

## 2 Methodology

This article is a narrative review examining the historical development, oral health effects, material properties, and environmental impact of CAT. A structured search of major biomedical and multidisciplinary databases, including PubMed/MEDLINE, Scopus, and Web of Science, was conducted, supplemented by manual screening of the reference lists from relevant publications. The search combined terms related to the review's main topics, such as “clear aligner therapy,” “orthodontic aligners,” “oral microbiome,” “biofilm,” “caries,” “periodontal health,” “thermoplastic polymers,” “polyurethane,” “PET-G,” “thermoforming,” “3D-printed aligners,” “microplastics,” “life cycle assessment,” and “environmental sustainability.” The literature reviewed ranged from foundational publications to the latest evidence available through 2025, aiming to cover both the origins of aligner therapy and recent advances in materials science and environmental research. Priority was given to peer-reviewed original articles, systematic reviews, meta-analyses, and relevant reference materials directly related to CAT or its biological, physicochemical, clinical, and ecological aspects. Publications were excluded if they were not directly relevant to clear aligner therapy, lacked sufficient scientific support, or did not substantially contribute to the goals of this interdisciplinary review.

## 3 Clear aligner therapy

### 3.1 Historical evolution of CAT

The evolution from fixed appliances to aligners represents incremental variations and improvements over the last 100 years, starting with the bracket-and-wire system and now progressing to polymer-based appliances [Table 1].

**Table 1:** History of CAT.

YEAR	MILESTONE	SIGNIFICANCE	REFERENCE
1945	Kesling introduces the tooth positioner concept for minor tooth movement using a removable appliance.	Establishes the foundational principle of staged tooth movement, which underlies modern aligner therapy.	(22)
1964	Nahoum develops a vacuum-formed thermoplastic appliance adapted to dental casts.	Introduces thermoformed plastic appliances that improve fit and retention compared to earlier concepts.	(22)
1971	Ponitz describes “invisible retainers” fabricated from clear thermoplastic material.	Provides early clinical documentation of clear removable appliances for orthodontic use.	(23)
1993	Sheridan popularizes Essix thermoplastic retainers in orthodontic practice.	Standardizes clear thermoplastic fabrication protocols, influencing later aligner workflows.	(24)
1997	Founding of Align Technology and introduction of CAD/CAM-based aligner concepts.	Marks the transition from artisanal appliances to digitally planned, mass-customized aligner therapy.	(25)
2011	Acquisition of Cadent (iTero intraoral scanning) by Align Technology.	Integrates digital impressions into aligner workflows, reducing dependence on physical casts.	(25)
2019	Development of biocompatible photopolymer resins for direct 3D printing of aligners.	Introduces direct-printed aligners, eliminating thermoforming and intermediate models.	(26)
2021	Publication of status reports on direct 3D printing of clear aligners.	Consolidates early evidence supporting feasibility and clinical interest in printed aligners.	(27)
2024	Clinical reports summarize design control and performance of direct-printed aligners.	Demonstrates maturation of additive manufacturing approaches in orthodontics.	(28)

Recently, younger generations of orthodontists have considered aligners to be the best technique for treating malocclusions (29). Nowadays, transparent aligners are manufactured with different polymers that are exposed to the intraoral environment, which comprises various substances, including water, electrolytes, enzymes, and bacteria, among other components (30). Additionally, changes in saliva acidity require a polymer that is chemically resistant, thermally stable, and volume- and mechanical-performance-invariant, to withstand occlusal forces and avoid fractures or deformations while maintaining elasticity and low hardness (29).

In recent years, the evolution of CAT has accelerated due to advances in digital dentistry, biomaterials science, and additive manufacturing. Contemporary aligner systems increasingly rely on fully digital workflows, including intraoral scanning, virtual treatment planning, and computer-aided manufacturing, thereby enhancing predictability and treatment efficiency. Notably, the introduction of directly 3D-printed aligners represents a paradigm shift by eliminating the need for intermediate physical models, potentially reducing material consumption and waste generation while maintaining clinically acceptable biomechanical performance. These developments highlight a transition from incremental design improvements toward system-level innovation in aligner fabrication (23–25).

### 3.2 Materials and modern manufacturing

Several thermoplastic materials, or combinations of polymers, are used for the manufacturing of these devices; these include polyvinyl chloride, polyurethane, polyethylene terephthalate, and polyethylene glycol terephthalate (31). Currently, the process to create a set of transparent aligners begins with virtual planning software using either initial plaster impressions that are subsequently scanned or direct intraoral scanning (27), the teeth's positions are manipulated through sequential movements towards the desired final positions, resulting in the generation of virtual models with teeth in different positions for each stage (29), A 3D physical model is needed for each individual aligner in the treatment set, and this is done through an additive or subtractive method, commonly 3D printing, stereolithography, or material injection (31). Three-dimensional printing with resin is currently the primary technique for fabricating orthodontic models (29). Subsequently, the corresponding series of transparent aligners are thermoformed on the physical copies and finally trimmed and polished. Depending on the type of malocclusion, the number of aligners added to the protocol for change and refinement determines the amount of plastic generated by the aligner treatment, which is becoming increasingly concerning (31).

Beyond aesthetics and patient comfort, aligner performance is governed by complex interactions between material properties, aligner geometry, thickness, and intraoral environmental factors. Recent studies emphasize that force delivery and decay are strongly influenced by viscoelastic behavior, water absorption, thermal fluctuations, and repeated insertion–removal cycles. Consequently, material selection and structural design have become central to aligner effectiveness, durability, and clinical predictability, prompting ongoing research into multilayered polymers and novel photopolymerizable resins (12, 14, 22).

Clinical performance of CAT is strongly influenced by the intrinsic properties of the polymers used and the manufacturing processes applied. In particular, thermoforming has been shown to induce significant alterations in aligner thickness, surface morphology, and mechanical behavior, which directly affect force delivery, stress relaxation, and clinical predictability (14). Comparative analyses of aligner thermoplastics further demonstrate that materials such as PET-G, thermoplastic polyurethanes, and polyurethane-based blends exhibit distinct viscoelastic responses and degradation patterns under thermal and aqueous aging conditions typical of the oral environment (12). In response to these limitations, recent studies have explored the direct three-dimensional printing of aligners using biocompatible photopolymerizable resins as an alternative manufacturing strategy, aiming to eliminate thermoforming-related variability while maintaining clinically acceptable mechanical properties (26, 32). Collectively, these findings underscore that material selection and fabrication method are central determinants of aligner effectiveness and constitute a critical link between orthodontic biomechanics and emerging sustainability considerations [Table 2].

**Table 2:** Materials of CAT.

MATERIAL	VERIFIED MATERIAL PROPERTIES	CLINICAL & ANALYTICAL IMPLICATIONS	REFERENCE
Polypropylene (PP)	Semi-crystalline thermoplastic with high stiffness and chemical resistance; limited transparency compared with PET-based materials.	Higher rigidity may improve strength but compromises esthetics; not a dominant modern aligner material.	(12)
Polycarbonate (PC)	Amorphous thermoplastic with good transparency, toughness, and dimensional stability.	Potential optical and mechanical advantages, but less commonly reported in current aligner systems.	(12)
PET-G	Transparent thermoplastic widely used in thermoformed aligners; thermoforming alters thickness, mechanical and surface properties.	Thermoforming-induced variability affects force delivery and fit, influencing clinical predictability.	(14)
Thermoplastic polyurethane (TPU)	Viscoelastic polymer exhibiting stress relaxation and sensitivity to thermal and aqueous aging.	Stress relaxation contributes to force decay over time, requiring careful material selection and design.	(12)
Polyurethane-based proprietary blends	Polyurethane systems optimized for elasticity and force consistency; performance influenced by thermoforming and intraoral aging.	Selected to improve sustained force delivery relative to other thermoplastics.	(22)
Multilayer polymer systems (PU or PETG/TPU composites)	Combine layers with different mechanical properties to modulate stiffness and elasticity.	Improve force consistency but complicate recycling and end-of-life management.	(22)
Photopolymerizable resins for direct 3D-printed aligners	Methacrylate/acrylate-based resins developed for biocompatible additive manufacturing of aligners.	Enable direct fabrication without thermoforming, improving geometric reproducibility but requiring strict post-curing protocols.	(26)

Recent evidence supports that multi-layer thermoformed materials have shown significantly better microscopic adaptation at the aligner–attachment interface than single-layer materials, and optimized attachment geometries also improved adaptation; importantly, no material–attachment combination achieved a perfect fit, underscoring that force transmission is materially and geometrically dependent rather than uniform across systems (33). Direct-printed aligners based on shape-memory resin have also demonstrated significantly higher and broader compressive-force ranges than thermoformed PETG controls, while allowing precise modification of inner thickness and local pressure areas, potentially enhancing selective biomechanical force delivery across different movement scenarios (34). In addition, thermo-responsive 4D aligners have been reported to retain their structural and functional properties after reshaping, with reversible changes in transparency occurring only under prolonged activation conditions that exceed the manufacturer's recommendations, supporting their thermo-mechanical stability within the tested protocol (35). Taken together, these findings suggest that aligners should not be considered mechanically interchangeable. Instead, the optimal CAT system for a given clinical case should be selected according to the specific physicochemical and mechanical requirements of treatment, including the need for conformability around attachments, the desired force range, and the expected response of the material under clinically relevant loading or thermal conditions (33, 34).

### 3.3 Effect of CAT on oral health

Aligners have shown to have a relatively minimal impact on the oral microbiome compared to other orthodontic appliances; however, changes have been observed in species such as *Porphyromonas gingivalis, Prevotella intermedia, Tannerella forsythia, Actinobacillus actinomycetemcomitans, Fusobacterium nucleatum* and *Treponema denticola* (36, 37). Nevertheless, the removable nature of aligners allows for better oral hygiene, as patients can easily clean both their teeth and the aligners. The ability to remove aligners during meals and oral care routines may result in a less disruptive impact on the oral microbiome than the continuous presence of fixed appliances (38, 39). However, it is important to note that aligner use still requires good oral hygiene practices, such as regular brushing and flossing, to prevent biofilm accumulation, as inadequate cleaning of aligners can also lead to bacterial growth and potentially cause oral health problems (40, 41). In this regard, microbial analysis of aligner-associated plaque has shown that clear aligners may harbor a diverse microbial community. Meto et al. reported the presence of oral microorganisms, including *Haemophilus parainfluenzae, Neisseria mucosa/sicca, Staphylococcus aureus, Streptococcus mitis/oralis, Streptococcus sanguinis*, and *Rothia aeria*, in biofilms associated with orthodontic clear aligners (42). Although this evidence derives from a case report and should be interpreted with caution, it reinforces the clinical relevance of effective aligner cleaning and disinfection protocols. These findings support the concept that the removable nature of aligners facilitates oral hygiene but does not eliminate the need for standardized maintenance strategies to reduce microbial plaque accumulation.

Recent systematic evidence suggests that, while clear aligners are generally associated with improved biofilm control and periodontal indices compared with fixed appliances, caries-related outcomes are more nuanced and appear to be strongly modulated by treatment duration and patient behaviors. Evidence comparing clear aligners with fixed orthodontic treatment reported differences in white spot lesions, plaque accumulation, and salivary caries-associated bacteria, highlighting that aligners can be advantageous for hygiene but do not eliminate cariogenic risk in susceptible patients (43). It has been shown that surface wear (microcracks/abrasions) and aligner irregularities can promote bacterial adherence and plaque biofilm, underscoring that aligner “removability” does not automatically translate into low microbiological risk without adequate hygiene (44). Recent microbiome-focused studies further support that both fixed appliances and aligners shift oral microbial communities, but aligner-associated changes tend to be more compatible with periodontal stability, albeit with measurable species-level fluctuations across treatment phases (45, 46). Importantly, experimental and clinical evidence indicate that aligner materials can harbor mixed biofilms, including *Streptococcus mutans* and *Candida albicans*, reinforcing the need for standardized cleaning protocols and patient instruction (47). Finally, emerging data warn that consuming acidic beverages without removing aligners may exacerbate enamel erosion/demineralization risk by creating a retentive acidic microenvironment, providing a clinically relevant rationale for reinforcing dietary guidance during aligner wear (48).

It has been shown that both fixed orthodontic appliances and CAT can alter the oral microbiome; however, the microbiological shifts observed during CAT appear to be more compatible with periodontal stability and better oral health than those commonly reported with fixed appliances (49). Nevertheless, these findings should not be interpreted as evidence of no biological risk, since aligner-associated microbial changes may still depend on oral hygiene, treatment duration, dietary habits, and material-related biofilm retention. In addition, recent evidence comparing PETG and TPU orthodontic thermoplastic materials suggests that aligner composition may influence time-dependent microbiome dynamics and salivary pH, highlighting the need to consider material-specific biological responses during clear aligner therapy (49, 50).

## 4 Environmental impact

The environmental impact of aligner waste has become a topic of growing concern in recent years (51). With the increasing popularity of aligners worldwide as an orthodontic treatment option, the amount of plastic waste generated by these devices has also increased (29, 52). Although aligners have several advantages and offer an efficient alternative to fixed appliances, we cannot overlook their disadvantages. Thus far, a particularly concerning drawback, about which there is limited literature, pertains to the environmental pollution they generate. These materials are predominantly petroleum-derived polymers characterized by high chemical stability and limited biodegradability, features that contribute to their persistence after disposal (53–55).

Recent studies emphasize that the environmental burden of CAT cannot be evaluated solely on the basis of visible plastic waste, but must be understood through a life cycle perspective encompassing raw material extraction, polymer synthesis, manufacturing, packaging, distribution, clinical use, and end-of-life disposal. Life cycle assessment (LCA) frameworks applied to plastic-based medical and dental devices show that single-use polymer systems can be associated with substantial cumulative environmental burdens, particularly when recycling or recovery pathways are limited (18, 56, 57). Within this framework, their management is further complicated because, after intraoral use, aligners may be treated as potentially contaminated healthcare waste depending on local regulatory frameworks and disposal practices (18, 56, 57). From a materials science standpoint, aligners are predominantly composed of petroleum-derived polymers characterized by high molecular weight, chemical stability, and resistance to environmental degradation. These properties, while advantageous for clinical durability, contribute to their persistence in the environment once discarded. Experimental and review-based evidence indicates that conventional disposal routes—such as landfilling and incineration—are associated with secondary environmental burdens, including microplastic generation, release of toxic by-products, and greenhouse gas emissions. Importantly, the multilayer and composite nature of many contemporary aligner materials further complicates recycling efforts, effectively excluding them from conventional plastic recovery streams (12, 17, 58).

Beyond solid waste considerations, recent research has raised concerns regarding the potential contribution of CAT to microplastic dissemination. *In vitro* and clinical studies suggest that mechanical wear, thermal cycling, and chemical exposure during aligner use can lead to the release of micro- and nanoscale polymer particles. While the clinical significance of oral microplastic exposure remains under investigation, their environmental relevance is well established, as microplastics are persistent pollutants that enter wastewater systems and broader ecological cycles. These findings extend the environmental discussion of aligners from post-disposal waste to in-use emissions, reinforcing the need for precautionary evaluation (59–61).

From a financial perspective, the global clear aligner market size was valued at $5.13 billion in 2023 and is projected to grow at a compound annual growth rate of 30.7% from 2024 to 2030. Additionally, the pandemic had a positive impact on global markets, and major aligner producers reported higher revenues in 2020 than in previous years (62). The rapid global expansion of the clear aligner market amplifies these environmental concerns by scaling polymer consumption and waste generation across millions of treatments annually. Market analysis projects sustained growth driven by adult demand and digital orthodontic workflows, implying that even modest per-patient material use translates into substantial aggregate environmental impact. In this context, orthodontists and manufacturers play a critical role in aligning clinical innovation with environmental responsibility. Recent interdisciplinary discussions advocate integrating sustainability principles into orthodontic practice, including waste segregation, patient education, material innovation, and exploring alternative manufacturing pathways, such as direct 3D printing, to reduce intermediate waste (63–65).

### 4.1 The CAT industry and its influence on the environment

Currently, the most commonly used thermoplastic materials in aligner manufacturing due to their ideal characteristics include modified polyethylene glycol terephthalate, polyester, polycarbonate, thermoplastic polyurethanes, polypropylene, and ethylene-vinyl acetate, as these materials enable high precision in three-dimensional thermoforming (66). These thermoplastic materials are non-biodegradable and can take many years to decompose in the environment, so they should not be disposed of in common waste (54). An example is polyethylene terephthalate, which belongs to the category of resistant plastics; it can take up to 1,000 years to decompose compared to a plastic bottle, which may take 450 years (55).

The current situation obliges us to reflect deeply on the importance of reducing the environmental impact of human activities. The Sustainable Development Goals (SDGs), defined by the United Nations in 2015, are guiding the way toward a society that is environmentally sustainable. Therefore, it is more essential than ever that, to achieve these important environmental goals for the community and future generations, all production sectors mobilize to innovate processes, practices, and products for use in their businesses (67). Dental care activities must be discussed since, for obvious reasons of hygiene, there are non-reusable wastes on a large scale and the use of chemicals that can cause harm to the environment (68).

CAT has become the preferred option for many orthodontists due to the high aesthetics and comfort they offer (69). One of the giants in the market, Align Technology, estimated 4 million cases treated in 2019 (70). In the economic sector, the global transparent aligner market in 2021 was projected at $3.1 billion USD, and it is estimated that by 2027, the figure will increase to $11.6 billion USD with an estimated annual growth of 13% (29). There are also at least 27 different companies manufacturing clear aligners and so-called in-office aligners (51). Collectively, these manufacturing pathways contribute to polymer waste generation and associated environmental burdens through model production, thermoforming, trimming losses, packaging, and distribution (71, 72).

Likewise, the manufacturing of aligners involves the use of other tools, processes, and materials, which can have an impact on the environment, among which the manufacturing process that requires 3D printing, generating non-reusable waste; the thermoplastic to make the aligners themselves that cannot be recycled; the series of studies, analyses, and designs that require working with computers. For this reason, the environmental impact of aligner production, as well as in all sustainability assessments, must be considered across various aspects (72).

To fully consider the environmental impact of aligners, the analysis should start from the indisputable consideration that the environmental profile of a given aligner system is context-dependent and may vary according to regional recycling infrastructure, waste-management practices, and the energy mix used during production and distribution (73–75) (for example, Germany, with a recycling rate of 55%) (73) as in a country where recycling rates are low or nonexistent. Mexico, for example, has a recycling rate of 5% (although it reaches values higher than 50% in the recycling of all PET consumed in the country) (74). The same consideration applies to the other equally important aspect when discussing the environmental impact of aligners, energy production. The process of study and production of the same treatment will have a lower environmental impact if carried out in a country like Costa Rica, where renewable energy production reaches 98% (mainly considering biomass, hydroelectric, geothermal, solar, and wind energy) (75) or if it is carried out in a country where energy production depends heavily on non-renewable sources. In a comprehensive assessment, then, something to consider is where the company is headquartered.

Another serious problem that has also been progressing is that after patients use aligners, they often dispose of them in regular waste, thus creating a risk of infection due to their use in the oral cavity. For this reason, aligners are considered as contaminated biomedical waste, making them unsuitable for recycling (76). Finally, burning plastic waste in incinerators can release chemicals and hazardous gases that severely damage the environment. Moreover, when harmful chemicals from burning, such as polychlorinated biphenyls and dioxins, are released into the atmosphere, they endanger all living beings (77).

Recent environmental analyses emphasize that the impact of clear aligner therapy should be interpreted through an LCA framework, rather than focusing exclusively on disposal. LCA-based evaluations of plastic medical and dental devices indicate that cumulative environmental burdens arise across multiple stages, including polymer synthesis, energy-intensive manufacturing, packaging, transportation, and end-of-life management. Importantly, studies highlight that single-use, patient-specific devices—such as CAT—exhibit disproportionately high environmental footprints when recycling pathways are limited or absent. Within this context, incineration, while commonly employed for contaminated healthcare plastics, may reduce biological risk but can contribute to greenhouse gas emissions and secondary pollutants if not performed under optimized conditions, underscoring the need for evidence-based disposal strategies rather than blanket assumptions of environmental benefit or harm (18, 56, 57).

In addition, recent sustainability frameworks for clear aligner therapy emphasize that mitigation should not rely exclusively on disposal-stage decisions, but also on upstream redesign and post-use recovery strategies (78). In practical terms, this includes the implementation of manufacturer or clinic-supported take-back programs for used aligners and associated packaging, which could facilitate safer segregation, recovery, repurposing, or specialized recycling pathways within a circular-economy model, even if conventional municipal recycling remains unsuitable for saliva-contaminated plastics (16, 17, 51, 78, 79). At the same time, the literature highlights the need for materials redesign, including the development of recyclable, bio-based, or lower-impact polymers, as well as alternative production workflows that reduce trimming waste and energy demand. Therefore, alongside LCA-based evaluation, future sustainability efforts in the aligner sector should integrate organized take-back systems and material innovation as complementary mitigation strategies (16, 17, 51, 78, 79).

### 4.2 Bioethical considerations in clear aligner therapy

Beyond clinical performance and material properties, CAT also raises relevant bioethical questions related to environmental responsibility, patient information, and the fair distribution of benefits and burdens associated with polymer-intensive orthodontic care. From a precautionary perspective, the environmental persistence and limited recyclability of many aligner materials support the need to minimize avoidable harm across the life cycle of these systems, from manufacturing and packaging to disposal and potential microplastic release (18, 56, 57). In this context, Hans Jonas’ ethics of responsibility provides a useful framework for considering the long-term consequences of technology-dependent healthcare interventions, particularly when environmental effects may extend beyond the immediate clinical setting (80, 81).

These concerns are also consistent with broader bioethical and sustainability frameworks. The UNESCO Universal Declaration on Bioethics and Human Rights emphasizes responsibilities toward present and future generations and highlights the interconnection between human beings, the environment, and other forms of life (82). Applied to CAT, this means that sustainability should not be treated as external to orthodontic care, but rather as part of responsible innovation, manufacturing, and clinical practice. Likewise, environmental justice is relevant because the economic benefits of a rapidly expanding aligner market and the environmental burdens associated with polymer production, waste generation, and disposal are not always distributed equitably across populations (62, 63, 83, 84).

From the standpoint of autonomy, patients may benefit from receiving clear information not only about treatment effectiveness and esthetic outcomes but also about material characteristics, disposal limitations, and sustainability-related considerations when relevant to decision-making. From the standpoint of non-maleficence, clinicians and manufacturers share a responsibility to reduce avoidable environmental burdens by improving waste segregation, supporting life-cycle-based assessment, and promoting material and manufacturing strategies that reduce unnecessary waste generation, including more efficient digital workflows and alternative production pathways when scientifically justified (56, 62–64, 68, 83, 84). In this sense, bioethical analysis complements clinical and materials-based evaluation by framing CAT as a treatment modality in which effectiveness, safety, and environmental responsibility should be considered together.

### 4.3 Alternatives for reducing the environmental impact of aligner production and usage

It is important to incorporate the 4R approach into plastic waste management, which involves reduction, reuse, recycling, and recovery (85). However, it is still necessary to explore the feasibility of applying this principle in orthodontics, particularly regarding aligners, which are considered biological waste due to their interaction with saliva and oral tissues. Technology has enabled the development of new production systems, such as the CAD-CAM system, a fully computerized process consisting of two phases. CAD stands for “computer-aided design,” and CAM stands for “computer-aided manufacturing” (86). The use of this system requires a large amount of electrical energy, which in most cases involves the combustion of fossil fuels (coal and oil), resulting in an increase in the carbon footprint. Therefore, future trends aim to transition to renewable sources (water, wind, solar) for aligner production. It is expected that in the future, major aligner companies will opt to integrate these types of systems into their production processes (87), and printing is done directly without the need to thermoform a material onto a model generated by digital printing (88). This would reduce the number of waste generated by aligner production (89) and it is also presumed that this system could present the advantage of being more precise because it would reduce errors generated during the thermoforming process (90).

From a materials perspective, recent studies highlight that the environmental challenge posed by CAT is compounded by the multilayer and composite nature of contemporary polymer systems, which are specifically engineered to optimize force delivery and mechanical performance (17, 91). While such designs enhance clinical effectiveness, they significantly limit recyclability by preventing efficient polymer separation. Moreover, comparative analyses between thermoformed and directly 3D-printed aligners suggest that manufacturing processes influence not only mechanical behavior but also environmental impact, as thermoforming introduces material-trimming waste and property variability, whereas direct printing may reduce intermediate waste streams at the cost of increased reliance on photopolymer resins. Additionally, material aging, mechanical wear, and exposure to the oral environment have been shown to promote surface degradation and microfragmentation, reinforcing concerns regarding both waste persistence and microplastic generation (17, 91).

## 5 Conclusions

Clear aligner therapy is a widely used orthodontic option because of its esthetic advantages, comfort, removability, and compatibility with digital workflows. Current evidence suggests that clear aligners may facilitate plaque control and support more favorable periodontal conditions than fixed appliances in selected patients. However, these benefits are not inherent to the appliance alone and depend strongly on patient behavior, oral hygiene adherence, dietary habits, treatment duration, baseline caries risk, and adequate aligner maintenance. Microbiological studies indicate that clear aligners do not eliminate the risk of biofilm accumulation, caries, enamel demineralization, or microbial shifts. Therefore, individualized risk assessment and patient education should remain central components of clear aligner therapy.

From a materials perspective, contemporary aligners rely mainly on thermoplastic and polyurethane-based polymers designed to provide transparency, elasticity, dimensional stability, and controlled force delivery. These properties are clinically advantageous but also contribute to environmental persistence and limited recyclability. Thermoforming, multilayer polymer systems, single-use trays, packaging, transport, and refinement protocols increase the environmental burden of CAT. Direct 3D-printed aligners may reduce intermediate model production and trimming waste, but they also raise unresolved questions about photopolymer resin composition, post-curing, energy demand, long-term biocompatibility, mechanical stability, and end-of-life management.

The environmental impact of clear aligners extends beyond disposal. Emerging evidence suggests that intraoral aging, mechanical loading, thermal changes, cleaning procedures, and chemical exposure may contribute to surface degradation and microplastic release during use. Although the clinical consequences of oral microplastic exposure remain under investigation, the environmental relevance of persistent polymer particles supports the need for precautionary and life-cycle-based evaluation. Addressing these concerns is challenging because used aligners may be saliva-contaminated, may not be accepted in conventional recycling systems, and often contain multilayer or composite polymers that are difficult to separate and recover.

Sustainable orthodontic practices should therefore combine immediate clinical actions with long-term innovations in materials and manufacturing. In the short term, orthodontists can contribute by reinforcing patient education, reducing unnecessary aligner replacements, improving treatment planning to minimize avoidable refinements, promoting appropriate cleaning practices, and encouraging responsible disposal or clinic-based collection when available. Manufacturers should support transparent life-cycle assessments, reduce packaging, optimize logistics, develop take-back programs, and invest in recyclable, bio-based, or lower-impact materials that do not compromise safety or clinical effectiveness. Future research should standardize methods to quantify environmental impact, microplastic release, and clinical performance across different aligner systems. In this way, clear aligner therapy can move toward sustainable orthodontic practices for current patients and future generations, balancing technological innovation, oral health, and environmental responsibility.
